# Supplementary material for: Anti-phage defence through inhibition of virion assembly
Source: Nat Commun. 2024 Feb 22;15:1644. doi: 10.1038/s41467-024-45892-x (PMC10884400; doi:10.1038/s41467-024-45892-x)
Supplement: Supplementary file 1 — Supplementary Information [file 41467_2024_45892_MOESM1_ESM.pdf]

Supplementary Information

Anti-phage defence through inhibition of virion assembly

Pramalkumar H. Patel[1], Véronique L. Taylor[1], Chi Zhang[2], Landon J. Getz[1], Alexa D. Fitzpatrick[1], Alan R. Davidson[1,2], and Karen L. Maxwell[1 *]

[1]Department of Biochemistry, University of Toronto, Ontario, Canada

[2]Department of Molecular Genetics, University of Toronto, Ontario, Canada

*Correspondence: karen.maxwell@utoronto.ca

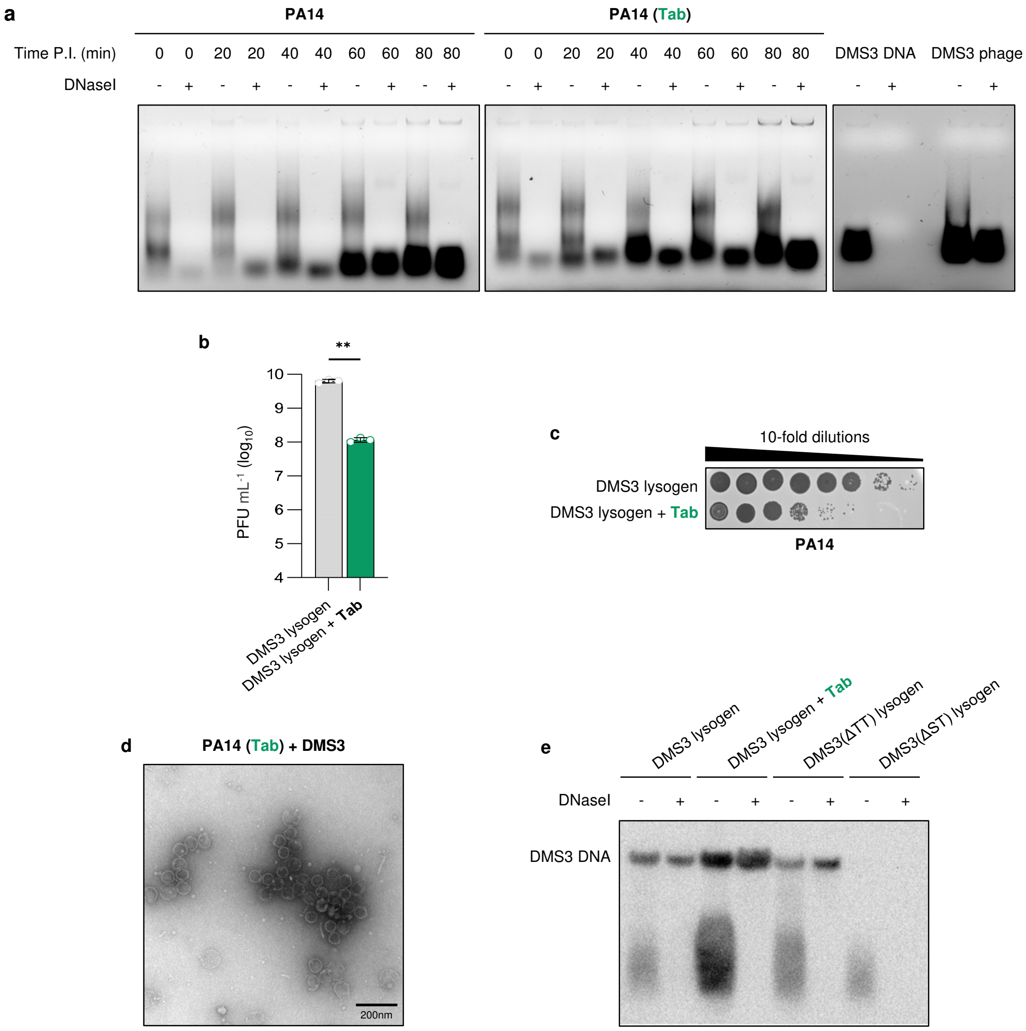

Supplementary Fig. 1: Tab inhibits virion formation during DMS3 prophage induction. a, Agarose gel showing the full gel used in the Southern blot presented in Fig.1d; total DNA was visualized by in-gel SYBR Safe staining. Time P.I., time post-infection. **b,** Assessing mitomycin C induced phage production in the absence and presence of Tab. Bars indicate the mean plaque-forming units per mL (PFU mL^{-1}), with the error bars representing the standard deviation (*n* = 3 biologically independent samples). ** *p*-value < 0.05 (paired *t*-test) between two groups; *p*-value = 0.0049 (DMS3 lysogen vs DMS3 lysogen + Tab). **c,** A representative image of the phage plating assay corresponding to panel **b**. **d,** A representative negatively stained transmission electron micrograph of a DMS3 lysate produced by infection of Tab-expressing cells. Scale bar, 200nm. Image is representative of three independent biological replicates. **e,** DNase protection assay of lysates produced by induction of DMS3 lysogens in the absence and presence of Tab, as well as induction of the DMS3 prophages with tail tube (TT) and small terminase (ST) single gene deletions. A Southern blot was used to assess the amount of phage DNA present in each sample.

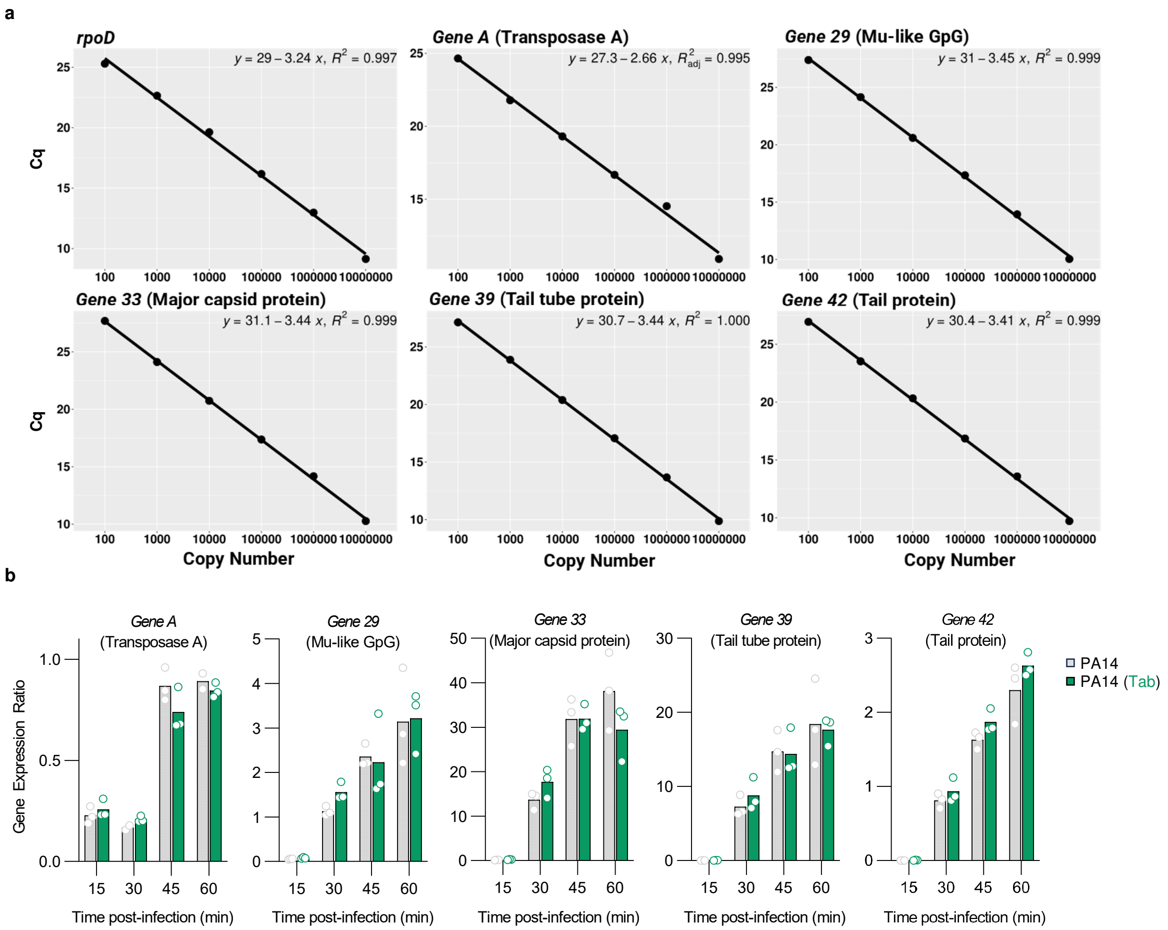

Supplementary Fig. 2: RT-qPCR standard curves and extended data. a, Copy number standard curves were generated to determine the relationship between Cq (quantification cycle) and transcript copy number for each primer set. This experiment was performed in triplicate and the Cq mean was calculated and plotted using R and the ggplot2 package. **b,** RT-qPCR was used to assess relative transcripts levels of early-expressed (*gene A*) and late-expressed genes (*29, 33, 39, and 42*). PA14 carrying an empty plasmid or a plasmid encoding Tab were infected with DMS3*vir* at an MOI of 2 and transcript copy number was measured by RT-qPCR. The expression of each phage gene was normalized to *rpoD* copy number and bars show mean gene expression ratio from three technical replicates. Data are representative of two biological replicates, with three technical replicates shown. Note: For *gene A* expression ratio, a total of two outlier data points were omitted for PA14 infection.

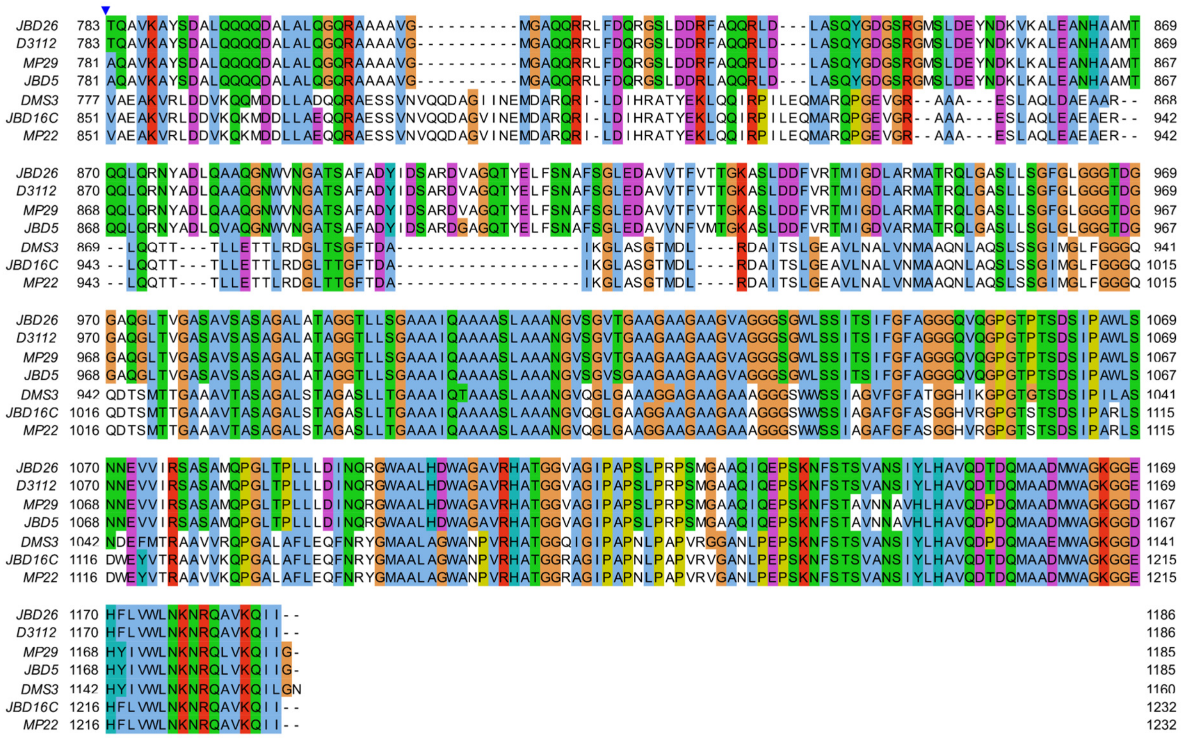

Supplementary Fig. 3: Sequence alignment of the C-terminal region of the Tape Measure Proteins. Sequence alignment was made using Jalview 2.11.2.7, and the residues colored using the ClustalX color scheme.

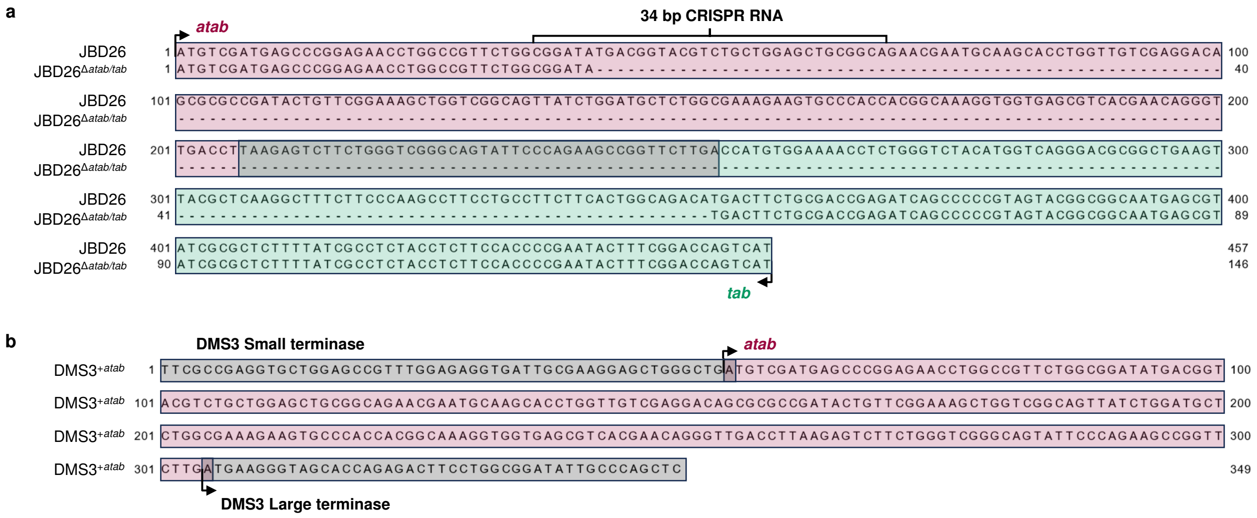

Supplementary Fig. 4: Sequences of JBD26$^{\Delta atab/tab}$ and DMS3^{+atab} mutants. a, Sequence alignment comparing WT phage JBD26 and phage JBD26 lacking *atab* and *tab*. The 271 base pair deletion in JBD26$^{\Delta atab/tab}$ is indicated by dashes and start sites (ATG) are shown by curved arrows. The CRISPR RNA (crRNA) target site is noted. The protein coding regions of *atab* and *tab* are colored in red and green, respectively. Sequences were aligned using Jalview 2.11.2.7. **b,** Sequence of phage DMS3^{+atab} (DMS3 containing anti-Tab from phage JBD26) in between the small and large terminase genes. The start sites of *atab* and the large terminase gene are denoted by curved arrows. The protein coding regions of the small and large terminase are shaded grey, while the *atab* coding region is shown in red.

Supplementary Table 1: List of bacterial and bacteriophage strains used in this study.

**Bacterial strains**	**Description**	**Source**
E. coli DH5α	Used for cloning; F– φ80lacZΔ M15 Δ (*lacZYA-argF*) *U169 recA1 endA1 hsdR17* (rK–mK+) *phoA supE44* λ- *thi–1 gyrA96 relA1*	New England Biolabs
E. coli BTH101	Used for BACTH; F^-, *cya-99*, *araD139*, *galE15, galK16*, *rpsL1* (Str^r), *hsdR2*, *mcrA1*, *mcrB1*	Euromedex
E. coli SM10 λ pir^+	Used for conjugation; *thi thr leu tonA lacY supE* recA::RP4-2-Tc::Mu	New England Biolabs
E. coli BL21(λDE3)	Used for protein expression; F– *omp*T $hsdS_B$ (r_B–, m_B–) *gal dcm* (DE3)	New England Biolabs
Pseudomonas aeruginosa strain UCBPP-PA14	UCBPP-PA14 strain	Human (origin - burn)
UCBPP-PA14 ΔCRISPR mutant	PA14 without a functional CRISRR-Cas system	[1]
UCBPP-PA14 *ΔpyoR/F* mutant	PA14 lacking R and F pyocins	[2]
PAO1	Lab stock of PAO1	Human (origin – burn)
PAO1 with wild-type I-C CRISPR	PAO1 with chromosomally integrated type I-C system, IPTG inducible	[3]
PAO1 I-$C^{\Delta csy3}$ system	PAO1 with chromosomally integrated type I-C helicase attenuated Cas3 system, IPTG inducible	[3]
PA14 DMS3(ΔST) lysogen (Clean in-frame deletion - Δ31-462 bp)	PA14 DMS3 lysogen lacking small terminase – Does not produce mature phage particles	This study
PA14 DMS3(ΔTT) lysogen (Clean in-frame deletion - Δ31-741 bp)	PA14 DMS3 lysogen lacking tail tube – Does not produce mature phage particles	This study
**Bacteriophages**		
JBD26	Wild-type phage; *Casadabanvirus*	[4]
DMS3	Wild-type phage; *Casadabanvirus*	[5]
DMS3*vir*	Lytic DMS3 variant - Truncated C-repressor protein	[1]
DMS3*m*	CRISPR-sensitive DMS3 phage	[6]
JBD24	Wild-type phage; *Casadabanvirus*	[4]
JBD16C	Wild-type phage; *Casadabanvirus*	[4]
JBD88a	Wild-type phage; *Casadabanvirus*	[4]
MP22	Wild-type phage; *Casadabanvirus*	[7]
JBD5	Wild-type phage; *Casadabanvirus*	[4]
MP29	Wild-type phage; *Casadabanvirus*	[8]
D3112	Wild-type phage; *Casadabanvirus*	[9]
LESφ4	Wild-type phage; *Casadabanvirus;* LESB58 prophage 4	[10]
JBD18	Wild-type phage; *Beetrevirus*	[4]
JBD25	Wild-type phage; *Beetrevirus*	[4]
YA3	Wild-type phage; unclassified *Caudoviricetes*	This study; Isolated independently fromcd [11]
Ab31	Wild-type phage; unclassified *Caudoviricetes*	[12]
JBD44a	Wild-type phage; unclassified *Caudoviricetes*	[4]
$DMS3^{+atab}$	DMS3 phage with *atab* from JBD26 phage (replacing *gp25* of DMS3*)*	This study
$JBD26^{\Delta atab/tab}$	JBD26 lacking *atab* and *tab*	This study
$DMS3^{6HisTT}$	DMS3 phage with histidine-tagged tail tube protein at the N-terminus	This study

DMS3$^{JBD16C\text{-}TMP}$	Chimeric DMS3 phage with TMP of JBD16C	This study
DMS3$^{JBD16C\text{-}TMP856}$	Chimeric DMS3 phage with the first 856 residues of JBD16C TMP	This study

TT = tail tube protein, TAC = tail assembly chaperone, TMP = Tape measure protein. Note: Phage YA3 was independently isolated and sequenced by C.Z and P.H.P.

Supplementary Table 2: List of plasmids used in this study.

Plasmids	Relevant features	Reference
pHERD30T	A shuttle vector that carries gentamycin resistant gene and pBAD promoter induced with L-(+)-arabinose	[13]
pHERD30T::*tab*[JBD26]	pHERD30T containing *tab* from phage JBD26 fused with a Flag-tag at the 3' end of the gene	This study
pKT25	BACTH vector with T25 fragment of *cya* gene	Euromedex
pKT25::*aqs1*	pKT25 with *aqs1* from DMS3	[14]
pKT25::*tab*	pKT25 with *tab* from phage JBD26	This study
pUT18C	BACTH vector with T18 fragment of *cya* gene	Euromedex
pUT18C::*pilB*	pUT18C with *pilB* from PA14	[14]
pUT18C::*atab*	pUT18C with *atab* from phage JBD26	This study
pHERD30T I-C	Empty pHERD30T I-C plasmid with modified I-C repeats	[3]
pHERD30T I-C:: *crRNA-JBD26-atab*	pHERD30T I-C vector containing crRNA targeting JBD26 *atab*	This study
pEXG2	Allelic exchange vector containing *sacB*, and gentamycin resistance cassette (Gm^R)	[15]
pEXG2::*DMS3*$^{+atab}$	Replacing DMS3 *gp25* with JBD26 *atab* construct	This study
pEXG2:: *DMS3_ Δsmallterminase*	Phage DMS3 small terminase deletion construct	This study
pEXG2:: *DMS3_ Δtailtubeprotein*	Phage DMS3 tail tube deletion construct	This study
pEXG2::*DMS3_NHis-tagged_tailtubegene*	Adding to N-terminal 6-His tag to tail tube protein of DMS3 construct	This study
pEXG2:: DMS3with16CTMP_swap	pEXG2 construct for swapping TMP of JBD16C into DMS3	This study
pETDuet-1	Vector for co-expressing two genes, pBR322-derived ColE1 replicon, *lacI* gene and ampicillin resistance gene, N-terminal 6-His tag in MCS site 1	Novagen (ID #71146)
pETDuet-1::6-His_Tab_site1	pETDuet-1 with *tab* at MCS site 1	This study
pETDuet1::6_His_Tab_site1 and_anti-Tab_site2	pETDuet-1 with *tab* at MCS site 1 and *atab* at MCS site 2	This study

Supplementary Table 3: List of oligonucleotide sequences used in this study.

**Primers**	**Sequence (5’ to 3’) and their descriptions in brackets**
**pHERD30T primers:**	
PHP10	AAAACCATGGCGACTGGTCCGAAAGTATTCGGGGTGG (Tab C-term FLAG-tag Forward)
PHP11	ATATAAGCTTTTACTTATCGTCGTCATCCTTGTAATCAGAGT (Tab C-term FLAG-tag Reverse)
	CTTCTGGGTCGGGCAGTATTCCCAG
**pHERD30T-derived I-C plasmid crRNA primers**	
PHP51	GCGACCGGATATGACGGTACGTCTGCTGGAGCTGCGGCAG
PHP52	GAAACTGCCGCAGCTCCAGCAGACGTACCGTCATATCCGG
**Bacterial two-hybrid primers:**	
PHP70	ATATTCTAGACATGACTGGTCCGAAAGTATTCGG (Tab in pKT25 F)
PHP71	ATATGGTACCTTAAGAGTCTTCTGGGTCGG (Tab in pKT25 R)
PHP72	ATATTCTAGACATGTCGATGAGCCCGGAGAACCTGG (Anti-Tab in pUT18C F)
PHP73	ATATGGTACCTCAAGAACCGGCTTCTGGGAATACTGCCCG (Anti-Tab in pUT18C R)
**pEXG2 construct primers:**	
pEXG2::ΔDMS3^{+atab}_swapping allele	
PHP91	TTTTCCATGGGCAGCTGCTCGCCAGCCTGG
PHP92	CGGCCAGGTTCTCCGGGCTCATCGACATCAGCCCAGCTCCTTCGCAATCACCTC
PHP93	AGGTGATTGCGAAGGAGCTGGGCTGATGTCGATGAGCCCGGAGAACCTGGCCG
PHP94	CGGGCAGTATTCCCAGAAGCCGGTTCTTGATGAAGAGTAGCACCAGGGACTTCCTGG
PHP95	CCAGGAAGTCCCTGGTGCTACTCTTCATCAAGAACCGGCTTCTGGGAATACTGCCCG
PHP96	TTTTAAGCTTGTGGAGCAGCGCATTACCGCG
**pEXG2::DMS3_NHis-tagged_tail tube protein mutant allele**	
PHP97	AAAAGAATTCTCTCGGCGACGAGATAGGCACCGG
PHP98	TTGCCCGTAGAAATACGTTTCCTGTGCGTGGTGGTGATGATGATGCATGTGAGGCCTCCGTAGGAATCAGATGTTGCG
PHP99	GCACAGGAAACGTATTTCTACGGGCAAGG
PHP100	AAAAGGTACCGCGGTGAACATGCCCACCTGGCGAG
**pEXG2::ΔDMS3_small terminase mutant allele**	
PHP101	TTTTAAGCTTTTGCTGGTGCCTACGGCGG
PHP102	CCAGCTCCTTCGCAATCACCTCTCCGGCGTCGCGGGTTTCCTTCGGGTG
PHP103	CACCCGAAGGAAACCCGCGACGCCGGAGAGGTGATTGCGAAGGAGCTGG
PHP104	TTTTGAATTCGACAGCGACCAACAAACTACTCGG
**pEXG2::ΔDMS3_tail tube protein mutant allele**	
PHP105	TTTTGGATCCGTGGCCGGCGCGGACGAGATCG
PHP106	CCAGCTGGATAATGCGACCGAAGCGTTGCCCGTAGAAATACGTTTCCTGTGCCATG
PHP107	CATGGCACAGGAAACGTATTTCTACGGGCAACGCTTCGGTCGCATTATCCAGCTGG
PHP108	TTTTAAGCTTTTCTCAGCAGTGATCGTGACTTTTACGG
**pEXG2::DMS3with16CTMP_swapping allele**	
PHP151	TAAAGCAAGCTTCTGCAGGTCGACTCTAGAGGATCCTTCGCTGACGGCGTGCTGAGCG
PHP152	AACCGCCAGGCCGTCAAGCAGATTATCTAGGAATTCATGGCTACTGAAATCGGCACCGCCACG
PHP153	AAAAGGTACCGGCCGGGTTGTTCGCTGTTCCTG
PHP154	AAAAGGATCCGATCCGCAGTATTACGCCCTGCGAG

PHP155	CAGTGTCAGTTGCTGATTGTTCGCCATCAGGATTCCAAATCGT TGAGGAAGGCGCTGG
PHP156	CCAGCGCCTTCCTCAACGATTTGGAATCCTGATGGCGAACAAT CAGCAACTGACACTG
**pETDuet-1 primers:**	
PHP160	ATATGGATCCGATGACTGGTCCGAAAGTATTCGGGGTGG (Tab MCS1 F)
PHP161	TTTTAAGCTTTTAAGAGTCTTCTGGGTCGGGCAGTATTCCCAG (Tab MCS1 R)
PHP162	ATATCATATGATGTCGATGAGCCCGGAGAACCTGG (Anti-Tab MCS2 F)
PHP163	ATATGGTACCTCAAGAACCGGCTTCTGGGAATACTGCCCG (Anti-Tab MCS2 R)
**Southern blotting primers:**	
PHP51	ATGATGGACTGGATTGCTGGTGCCTACG (DMS3 *gp25* F)
PHP52	CTATTCGCTGAAGGGGTCGTACCGACTG (DMS3 *gp25* R)
PHP53	ATGTCCGGAAAAGCGCAACAGCAATCTCG (PA14 *rpoD* F)
PHP54	ATTTCCTTGATCTCGGCGACGGTCAG (PA14 *rpoD* R)
**RT-qPCR primers:**	
PHP121	ATGGCACAGGAAACGTATTTCTACGGGCAAGG (DMS3 Tail tube Standard F)
PHP122	TTACCCCAGCTGGATAATGCGACCGAAGCGG (DMS3 Tail tube Standard R)
LG76	GTCGAGCACAAGGAAAGCTACA (DMS3 Tail Tube qPCR F1)
LG77	CCTTAGTCACGACCTTGCCATAG (DMS3 Tail Tube qPCR Reverse)
PHP123	ATGGCTACTGAAATCGGCACCGCCACG (DMS3 Tail protein Standard F)
PHP124	TCAGTCCTTCCTGATCGCGTAGAGCCAAGG (DMS3 Tail protein Standard R)
LG78	AAGCAACGAGTCCGACTACAACAA (DMS3 Tail Protein qPCR Forward)
LG79	AGCGGGAACTTGCTGAAAGAGT (DMS3 Tail Protein qPCR Reverse)
PHP125	ATGGCCATCATTACTCCGGCGCTGATTAGCG (DMS3 Major Capsid Standard F)
PHP126	TCAGTTGAGCCAGGCGGTATCGAGTACCTGG (DMS3 Major Capsid Standard R)
LG80	AACCTCGTTCCAGAAGCATTTCC (DMS3 Major Capsid qPCR F2)
LG81	CAGCCAGCCATAGGTGTTGC (DMS3 Major Capsid qPCR R2)
**Primers used below have been published**[14,16]	
PA14_rpoD_extF	GAGATGCGGTTGAGCTTGTT (rpoD Standard F)
PA14_rpoD_extR	GTCGACAGCGTCCTGAAGAG (rpoD Standard F)
PA14_rpoD_intF4	GGGCGAAGAAGGAAATGGTC (rpoD qPCR Forward)
PA14_rpoD_intR4	CAGGTGGCGTAGGTAGAGAA (rpoD qPCR Reverse)
DMS3gpA_extF	ATGGGTAGAGGCGGTGGG (gpA Standard F)
DMS3gpA_extR	TCATGCTTGCCGCTGCT (gpA Standard R)
DMS3gpA_intF3	GTACCACATTTCCAGCCTAC (gpA qPCR F)
DMS3gpA_intR3	CGGTTGGTTTCGACCAG (gpA qPCR R)
DMS3gpG_extF	GTGACAACTAGGATCGACGTCG (gpG Standard F)
DMS3gpG_extR	CTACCGATTTCGGCTCAAGG (gpG Standard R)
DMS3gpG_intF4	GGACGACCAGGAGGTTC (gpG qPCR F)
DMS3gpG_intR4	GTCCATAAAGGCGAACTCAG (gpG qPCR R)

Supplementary References:

1. Cady, K. C., Bondy-Denomy, J., Heussler, G. E., Davidson, A. R. & O'Toole, G. A. The CRISPR/Cas Adaptive Immune System of *Pseudomonas aeruginosa* Mediates Resistance to Naturally Occurring and Engineered Phages. *Journal of Bacteriology* **194**, 5728–5738 (2012).
2. Ojobor, C. D. The Noncontractile Phage Tail-like Bacterial Killing Nanomachines – Characterizing the Specificity Determinants of the F-Pyocins of *Pseudomonas aeruginosa*. (2022).
3. Csörgő, B. *et al.* A compact Cascade–Cas3 system for targeted genome engineering. *Nat Methods* **17**, 1183–1190 (2020).
4. Bondy-Denomy, J. *et al.* Prophages mediate defense against phage infection through diverse mechanisms. *ISME J* **10**, 2854–2866 (2016).
5. Zegans, M. E. *et al.* Interaction between Bacteriophage DMS3 and Host CRISPR Region Inhibits Group Behaviors of *Pseudomonas aeruginosa*. *Journal of Bacteriology* **191**, 210–219 (2009).
6. Bondy-Denomy, J., Pawluk, A., Maxwell, K. L. & Davidson, A. R. Bacteriophage genes that inactivate the CRISPR/Cas bacterial immune system. *Nature* **493**, 429–432 (2013).
7. Heo, Y.-J., Chung, I.-Y., Choi, K. B., Lau, G. W. & Cho, Y.-H. Genome sequence comparison and superinfection between two related *Pseudomonas aeruginosa* phages, D3112 and MP22. *Microbiology,* **153**, 2885–2895 (2007).
8. Chung, I.-Y. & Cho, Y.-H. Complete genome sequences of two *Pseudomonas aeruginosa* temperate phages, MP29 and MP42, which lack the phage-host CRISPR interaction. *J Virol* **86**, 8336 (2012).
9. Wang, P. W., Chu, L. & Guttman, D. S. Complete Sequence and Evolutionary Genomic Analysis of the *Pseudomonas aeruginosa* Transposable Bacteriophage D3112. *J Bacteriol* **186**, 400–410 (2004).
10. Winstanley, C. *et al.* Newly introduced genomic prophage islands are critical determinants of in vivo competitiveness in the Liverpool Epidemic Strain of *Pseudomonas aeruginosa*. *Genome Res* **19**, 12–23 (2009).
11. Yu, X. *et al.* Molecular Characterization and Comparative Genomic Analysis of vB_PaeP_YA3, a Novel Temperate Bacteriophage of *Pseudomonas aeruginosa*. *Frontiers in Microbiology* **11**, (2020).
12. Latino, L., Essoh, C., Blouin, Y., Thien, H. V. & Pourcel, C. A novel *Pseudomonas aeruginosa* Bacteriophage, Ab31, a Chimera Formed from Temperate Phage PAJU2 and P. putida Lytic Phage AF: Characteristics and Mechanism of Bacterial Resistance. *PLOS ONE* **9**, e93777 (2014).
13. Qiu, D., Damron, F. H., Mima, T., Schweizer, H. P. & Yu, H. D. PBAD-Based Shuttle Vectors for Functional Analysis of Toxic and Highly Regulated Genes in *Pseudomonas* and *Burkholderia spp.* and Other Bacteria. *Applied and Environmental Microbiology* **74**, 7422–7426 (2008).
14. Shah, M. *et al.* A phage-encoded anti-activator inhibits quorum sensing in *Pseudomonas aeruginosa*. *Molecular Cell* **81**, 571-583.e6 (2021).
15. Rietsch, A., Vallet-Gely, I., Dove, S. L. & Mekalanos, J. J. ExsE, a secreted regulator of type III secretion genes in *Pseudomonas aeruginosa*. *Proceedings of the National Academy of Sciences* **102**, 8006–8011 (2005).

16. Stanley, S. Y. *et al.* Anti-CRISPR-Associated Proteins Are Crucial Repressors of Anti-CRISPR Transcription. *Cell* **178**, 1452-1464.e13 (2019).
