# Supplementary material for: Anti-phage defence through inhibition of virion assembly
Source: Nat Commun. 2024 Feb 22;15:1644. doi: 10.1038/s41467-024-45892-x (PMC10884400; doi:10.1038/s41467-024-45892-x)
Supplement: Supplementary file 3 — Description of Additional Supplementary Files [file 41467_2024_45892_MOESM3_ESM.pdf]

Description of Additional Supplementary Files:

Supplementary Data 1: Full nucleotide sequences of phages $DMS3^{16C\text{-}TMP}$ and $DMS3^{16C\text{-}TMP856}$.
