# Supplementary material for: Anti-phage defence through inhibition of virion assembly
Source: Nat Commun. 2024 Feb 22;15:1644. doi: 10.1038/s41467-024-45892-x (PMC10884400; doi:10.1038/s41467-024-45892-x)
Supplement: Supplementary file 5 — Reporting Summary [file 41467_2024_45892_MOESM5_ESM.pdf]

nature portfolio

Corresponding author(s): Karen L. Maxwell

Last updated by author(s): Jan 13, 2024

Reporting Summary

Nature Portfolio wishes to improve the reproducibility of the work that we publish. This form provides structure for consistency and transparency in reporting. For further information on Nature Portfolio policies, see our Editorial Policies and the Editorial Policy Checklist.

Statistics

For all statistical analyses, confirm that the following items are present in the figure legend, table legend, main text, or Methods section.

n/a	Confirmed	
[ ]	[x]	The exact sample size (*n*) for each experimental group/condition, given as a discrete number and unit of measurement
[ ]	[x]	A statement on whether measurements were taken from distinct samples or whether the same sample was measured repeatedly
[ ]	[x]	The statistical test(s) used AND whether they are one- or two-sided<br>*Only common tests should be described solely by name; describe more complex techniques in the Methods section.*
[x]	[ ]	A description of all covariates tested
[x]	[ ]	A description of any assumptions or corrections, such as tests of normality and adjustment for multiple comparisons
[ ]	[x]	A full description of the statistical parameters including central tendency (e.g. means) or other basic estimates (e.g. regression coefficient) AND variation (e.g. standard deviation) or associated estimates of uncertainty (e.g. confidence intervals)
[ ]	[x]	For null hypothesis testing, the test statistic (e.g. *F*, *t*, *r*) with confidence intervals, effect sizes, degrees of freedom and *P* value noted<br>*Give P values as exact values whenever suitable.*
[x]	[ ]	For Bayesian analysis, information on the choice of priors and Markov chain Monte Carlo settings
[x]	[ ]	For hierarchical and complex designs, identification of the appropriate level for tests and full reporting of outcomes
[x]	[ ]	Estimates of effect sizes (e.g. Cohen's *d*, Pearson's *r*), indicating how they were calculated

Our web collection on statistics for biologists contains articles on many of the points above.

Software and code

Policy information about availability of computer code

Data collection	TECAN growth curves data were collected using TECAN Infinite 200 instrument with Magellan software. AMT Image Capture Engine Software Version 602.492 was used to image transmission electron micrographs. Bio-Rad CFX384 Real-Time 384-well PCR qPCR Detection System was used to perfom RT-qPCR experiments. NCBI PSIblast was used to perform protein sequence searches (June 2023).
Data analysis	GraphPad Prism 9.0.2 was used for graphing data and performing statistical analysis. Jalview 2.11.2.7 was used to make alignment figures. Clinker v0.0.28 was used to make gene cluster figures. Bio-Rad CFX Maestro 1.1 (version 4.1.2433.1219) was used to analyze RT-qPCR data. RStudio version 4.3.1, ggplot version 3.4.4 and ggpmisc version 0.5.5 packages were used to plot RT-qPCR data. NucleAIzer was used to segment heads, proheads and capsids, and their area calculations were performed using ImageJ version 1.54.

For manuscripts utilizing custom algorithms or software that are central to the research but not yet described in published literature, software must be made available to editors and reviewers. We strongly encourage code deposition in a community repository (e.g. GitHub). See the Nature Portfolio guidelines for submitting code & software for further information.

Data

Policy information about availability of data

All manuscripts must include a data availability statement. This statement should provide the following information, where applicable:

- Accession codes, unique identifiers, or web links for publicly available datasets
- A description of any restrictions on data availability
- For clinical datasets or third party data, please ensure that the statement adheres to our policy

Data that support the findings of this study are available within the article, Supplementary Information, Supplementary Data or the Source Data file. Primer sequences are available in Supplementary Table 3. Raw sequencing files associated with Fig. 3c have been uploaded as FASTA files in Supplementary Data 1. Source data are provided with this manuscript.

Research involving human participants, their data, or biological material

Policy information about studies with human participants or human data. See also policy information about sex, gender (identity/presentation), and sexual orientation and race, ethnicity and racism.

Reporting on sex and gender	Not applicable
Reporting on race, ethnicity, or other socially relevant groupings	Not applicable
Population characteristics	Not applicable
Recruitment	Not applicable
Ethics oversight	Not applicable

Note that full information on the approval of the study protocol must also be provided in the manuscript.

Field-specific reporting

Please select the one below that is the best fit for your research. If you are not sure, read the appropriate sections before making your selection.

[x] Life sciences [ ] Behavioural & social sciences [ ] Ecological, evolutionary & environmental sciences

For a reference copy of the document with all sections, see nature.com/documents/nr-reporting-summary-flat.pdf

Life sciences study design

All studies must disclose on these points even when the disclosure is negative.

Sample size	All experiments were performed in triplicates (n =3 or higher) for robust statistical analyses. This sample size was sufficient to detect similarities or differences between different samples and conditions. Samples sizes for EM experiments were determined based on instrument, resource availability, and consistent phenotypes. For measuring capsid area in Fig.2e, consistent range of phenotypes were measured for 135 capsids, and the results are documented in the Source Data file.
Data exclusions	During the RT-qPCR experiments, a total of two outlier data points related to gene A expression ratio in PA14 cells infected with DMS3 at 30 and 60 minute times were removed from the analysis. These data are provided in the Source Data file and highlighted. Apart from these instances, no other data were omitted in the study.
Replication	Other than RT-qPCR experiments (performed in duplicates), all experiments were performed in at least three independent biological replicates. All reported data were successfully reproduced.
Randomization	Randomization was not necessary for the experiments performed since groups were not allocated.
Blinding	Blinding was not necessary to the study as biases or expectations of the experimenter were not anticipated to influence the experimental results.

Reporting for specific materials, systems and methods

We require information from authors about some types of materials, experimental systems and methods used in many studies. Here, indicate whether each material, system or method listed is relevant to your study. If you are not sure if a list item applies to your research, read the appropriate section before selecting a response.

Materials & experimental systems

n/a	Involved in the study
☐	☒ Antibodies
☒	☐ Eukaryotic cell lines
☒	☐ Palaeontology and archaeology
☒	☐ Animals and other organisms
☒	☐ Clinical data
☒	☐ Dual use research of concern
☒	☐ Plants

Methods

n/a	Involved in the study
☒	☐ ChIP-seq
☒	☐ Flow cytometry
☒	☐ MRI-based neuroimaging

Antibodies

Antibodies used	1. Mouse 6x-His Tag monoclonal antibody (TAG001.100) (BioShop) at 1:5000 for Western blots<br>2. Horse Anti-mouse IgG, HRP-linked Secondary Antibody (Cell Signalling Technology #7076) at 1:10000 for Western blots<br>3. Anti-Digoxin–Alkaline Phosphatase antibody, Mouse monoclonal clone DI-22, purified from hybridoma cell culture (Roche; 11333062910) at 1:10000 for Southern blots
Validation	1. Manufacturer's website: "High Specificity, High Affinity (1-5ng tagged protein) Recognizes both N- and C- terminal tag. Tested in Western blot, IP and IC. Supplied in PBS solution, 1mg/ml. Mouse monoclonal (IgG2b)."<br>2. Manufacturer's website: "This product is thoroughly validated with CST primary antibodies and will work optimally with the CST western immunoblotting protocol, ensuring accurate and reproducible results."<br>3. Validation in Roche DIG Application Manual for Filter Hybridization page 24.
